# Impact of 4 different definitions used for the assessment of the prevalence of the Metabolic Syndrome in primary healthcare:The German Metabolic and Cardiovascular Risk Project (GEMCAS)

**DOI:** 10.1186/1475-2840-6-22

**Published:** 2007-09-06

**Authors:** Susanne Moebus, Jens Ulrich Hanisch, Pamela Aidelsburger, Peter Bramlage, Jürgen Wasem, Karl-Heinz Jöckel

**Affiliations:** 1 Institute for Medical Informatics, Biometry and Epidemiology, University Hospital, Essen, Germany; 2 CAREM GmbH, Sauerlach, Germany; 3 Institute for Clinical Pharmacology, Medical Faculty Carl-Gustav Carus, TU Dresden, Germany; 4 Alfried Krupp von Bohlen und Halbach Foundation, Institute for Healthcare Management, University of Duisburg-Essen, Germany

## Abstract

**Background:**

The metabolic syndrome (MetSyn) places individuals at increased risk for type 2 diabetes and cardiovascular disease. Prevalence rates of the population of the MetSyn are still scarce. Moreover, the impact of different definitions of the MetSyn on the prevalence is unclear. Aim here is to assess the prevalence of the MetSyn in primary health care and to investigate the impact of four different definitions of the MetSyn on the determined prevalence with regard to age, gender and socio-economic status.

**Methods:**

The German-wide cross-sectional study was conducted during two weeks in October 2005 in 1.511 randomly selected general practices. Blood samples were analyzed, blood pressure and waist circumference assessed, data on lifestyle, medication, chronic disorders, and socio-demographic characteristics collected. MetSyn prevalence was estimated according to the definitions of NCEP ATP III (2001), AHA/NHLBI (2004, 2005), and IDF (2005). Descriptive statistics and prevalence rate ratios using the PROG GENMOD procedure, were calculated. Cohen's kappa was used as measure for interreliability between the different prevalence estimates.

**Results:**

Data of 35,869 patients (age range: 18–99, women 61.1%) were included. The prevalence was lowest using the NCEP ATP III- (all: 19.8%, men 22.7%, women: 18.0%), highest according to the IDF-definition (32.7%, 40.3%, 28.0%). The increase in prevalence with recent definitions was more pronounced for men than for women, and was particularly high for men and women aged 60–79 years. The IDF-definition resulted in a higher prevalence especially in those with the highest educational status. Agreement (kappa) between the NCEP ATP III- and IDF-definition was 0.68 (men 0.61, women 0.74), between the updated the AHA/NHLBI- (2005) and IDF-definition 0.85 (men 0.79, women 0.89).

**Conclusion:**

The prevalence of metabolic syndrome is associated with age, gender, and educational status and increases considerably with each newly published definition. Our data highlight the need for a better evidence regarding thresholds of the components of the metabolic syndrome, especially with regard to the IDF-definition – according to which in some populations a majority of subjects are diagnosed with the metabolic syndrome.

## Background

Over the past decade the Metabolic syndrome (MetSyn) has been granted increased attention due to its postulated impact on cardiovascular diseases, especially if linked to the increasing problem of (central) adiposity.

There are many different terms – Syndrome X, Deadly Quartet, Plurimetabolic Syndrome, Insulin Resistance Syndrome, or recently Cardiometabolic Syndrome – for the Metabolic syndrome and many faces of the Metabolic Syndrome, since almost every other year in the last 8 years a new definition for diagnosis of MetSyn has been released (Tab. 1). The most widely used is the National Cholesterol Education Program Adult Treatment Panel III (NCEP ATP III) definition [1]. In contrast to the definition of the World Health Organization (WHO) [2], it does not require the determination of insulin levels, thereby facilitating prevalence assessment. In 2004 and 2005, the NCEP ATP III-definition has been modified by the American Heart Association and the National Heart, Lung, and Blood Institute (AHA/NHLBI) [3,4]. In 2005, the major adjustment to the AHA/NHLBI 2004 definition was to include persons reporting a history of current antihypertensive drug or lipid lowering medication use regardless of measured values (Tab. 2).

**Table 1:** Timeline of most popular definitions of the Metabolic Syndrome

**Year**	**Organization**
1998	WHO, World Health Organization
1999	EGIR (European Group for the Study of Insulin Resistance)
2001	NCEP ATP III (National Cholesterol Education Program/Adult Treatment Panel)
2002	ACE (American College of Endocrinology)
2004	AHA/NHLBI (National Heart, Lung, and Blood Institute/American Heart Ass.)
2005	IDF (International Diabetes Federation)
2005	AHA/NHLBI (National Heart, Lung, and Blood Institute/American Heart Ass.)

**Table 2:** Exact NCEP ATP-III, AHA/NHLBI and IDF-definitions of the Metabolic Syndrome as originally published

**Risk factor**	**NECP ATP III** 2001 [1]*	**AHA/NHLBI** **2004 [3]^†^**	**AHA/NHLBI** **2005 [4]^†^**	**IDF** 2005 [5]**
**Central/Abdominal obesity (Waist circumference)**	M >102 cm^‡^ W > 88 cm ^‡^	M >102 cm ^‡^ W > 88 cm ^‡^	M ≥ 102 cm ^§,||^ W ≥ 88 cm ^§,||^	**Must have**: Central obesity, defined as WC^¶ ^**with ethnicity specific values. Europids: male ≥ 94 cm, female ≥ 80 cm plus any two of following 4 factors:**
**Blood pressure**	≥130/≥85 mmHg	≥130/≥85 mmHg	≥130 mm Hg systolic BP, or ≥ 85 mm Hg diastolic BP, **or drug treatment for hypertension**	≥130 mm Hg systolic blood pressure, or ≥ 85 mm Hg diastolic blood pressure, **or treatment of previously diagnosed hypertension**
**Fasting glucose**	110 mg/dL^#^	110 mg/dL**	**≥100 mg/dL or drug treatment for elevated glucose**	**≥5.6 **mmol/L (100 mg/dL) or **previously diagnosed diabetes. If above 5.6 mmol/L, OGTT is strongly recommended but is not necessary to define presence of the syndrome**
**Triglycerides**	150 mg/dL	150 mg/dL	≥150 mg/dL (1.7 mmol/L) **or drug treatment for elevated TG^††^**	≥1.7 mmol/L (150 mg/dL) **or specific treatment for this lipid abnormality **
**HDL cholesterol**	M <40 mg/dL W <50 mg/dL	M <40 mg/dL W<50 mg/dL	M <40 mg/dL (0.9 mmol/L)^†† ^W <50 mg/dL (1.1 mmol/L)^†† ^**or drug treatment for reduced HDL-C**^‡‡^	M <1.03 mmol/L (40 mg/dL) W <1.29 mmol/L (50 mg/dL) **or specific treatment for this lipid abnormality**

Differences relative to the NCEP ATP III definition are shown in bold ; M = men, W = women

* sometimes referred to as [44]

^† ^often cited as NCEP ATP III definition

**see also [6]

^§ ^changed from ">" to "≥"

† † erratum in: Circulation. 2005 Oct 25;112(17):e297 and e298 (1.03 and 1.3 mmol/L) for apparently wrong original published values;

Following remarks are all citations of the original definitions:

‡ Overweight and obesity are associated with insulin resistance and the metabolic syndrome. However, the presence of abdominal obesity is more highly correlated with the metabolic risk factors than is an elevated BMI. Therefore, the simple measure of WC is recommended to identify the body weight component of the metabolic syndrome

|| Lower WC cut point (eg, ≥ 90 cm in men and ≥ 80 cm in women) appears to be appropriate for Asian Americans

¶if BMI is >30, central obesity can be assumed and waist circumference does not need to be measured

# "... the presence of type 2 diabetes does not exclude a diagnosis of metabolic syndrome."

** "The ADA has recently established a cutpoint of ≥ 100 mg/dL, above which persons have either prediabetes (impaired fasting glucose) or diabetes. This new cutpoint should be applicable for identifying the lower boundary to define an elevated glucose as one criterion for the metabolic syndrome"

‡‡ Fibrates and nicotinic acid are the most commonly used drugs for elevated TG and reduced HDL-C. Patients taking 1 of these drugs presumed to have high TG and low HDL.

Also in 2005, and prior to the release of the 2005 revision of the AHA/NHLBI-definition, the International Diabetes Federation (IDF) announced a new, meant to be globally applicable "platinum standard" definition of the MetSyn [5]. The IDF sharply reduced the cutoff value for central adiposity and made central adiposity as a prerequisite to the diagnosis of the MetSyn [6]. One of the rationale of this definition was to provide a standard definition that could enable better comparisons between studies with a standardized clinical diagnose.

The use of different definitions might have an impact on the determined prevalence and confuses the interpretation of epidemiological studies. While earlier studies on the prevalence of the MetSyn primarily used the WHO and EGIR definitions, most recent studies have used the NCEP ATP III-definition.

An aggravating factor is that to date populationwide data about precise frequencies and distributions of the MetSyn occurrence is still missing and only few studies have used the IDF-definition to estimate the prevalence of the MetSyn [7]. Furthermore, to our knowledge, there is only few data available, showing the effect of more than two definitions on the prevalence of the MetSyn in the population, especially with their possible impact of different subgroups.

The German metabolic and cardiovascular risk project (GEMCAS) is based on a nationwide prevalence study to assess the MetSyn in a primary care patient population, with emphasis on the young (<40 years) and elderly (>75), an often disregarded population group in epidemiological and clinical studies. The aim of this study was (1) to assess the prevalence of the Metabolic syndrome in Germany in a primary health care setting, (2) to assess the impact of the NCEP ATP III-, AHA/NHLBI- and IDF-definitions on the overall prevalence rate, and (3) to determine the impacts of these definitions on prevalence rates with regard to age, gender and educational status.

## Methods

GEMCAS, a cross-sectional study, was conducted during two weeks in October 2005 at 1,511 randomly selected general health care practices across Germany. Practitioners specialized in cardiology and/or diabetes were excluded to prevent results biased toward false higher prevalence, since especially patients with diabetes and CVD attend these physicians.

Study methods have been described in detail [8]. In short, all eligible patients aged ≥ 18 years visiting a general practitioner were included in the study population if they gave their written informed consent. The study protocol was approved by the institutional ethics committee.

All parameters required for diagnosis of MetSyn were assessed: waist circumference, blood pressure, glucose, triglyceride and HDL-cholesterol levels. To account for the effect of fasting status, a 2-step approach was used. A blood glucose quick test was performed to distinguish diabetic (>11.1 mmol/L) and nondiabetic (<5.58 mmol/L) patients even under nonfasting conditions. This was done to minimize the need for follow-up appointments, as only 17% of all participants were able to provide a fasting blood sample on the survey day. However, blood glucose concentrations between 5.56–11.1 mmol/L in the quick test required a follow up fasting blood sample. Additionally, to assess intra-individual variability, 30% of the study population was scheduled for a follow-up visit irrespective of the result of their blood glucose quick test. All blood samples were analyzed by a central laboratory (Berlin, Germany).

Data were collected on sociodemographic variables, smoking habits, lifestyle aspects and on a history of cardiovascular disease (CVD) and diabetes. For quality assurance, a monitoring concept including telephone-monitoring and selective random on-site monitoring was performed. Non-responder information were collected including age, sex, obesity, reason for visiting the general practitioner, and reason for not participating.

To evaluate the impact of different criteria and cutoff values for diagnosing the MetSyn, the prevalence was assessed using NCEP ATP III-, AHA/NHLBI- and IDF-definitions. Since numerous studies using the NCEP ATP III-definition included hypertensive treatment, although explicitly not mentioned in the original definition (Tab. 2), we additionally considered subjects using hypertensive medication as having MetSyn (modified NCEP ATP III-definition).

Descriptive statistics (mean, standard deviation, 95%-confidence interval) and prevalence rate ratios (PRR), using PROG GENMOD procedure, were calculated. Cohen's kappa was used as measure for interreliability between the different prevalence estimates. As the samples were clustered with respect to the physicians offices reporting the health status of several patients, the Taylor expansion method was used for analysis, applying the SAS procedures SURVEYFREQ and SURVEYMEANS.

## Results

### Characteristics of the study population

The study population has been described in detail [8]. Briefly, 35,869 patients (38.9% men, 61.1% women), age range 18–99 years were included in the analysis. Mean age was 53.0 ± 15.8 years for men, women 51.9 ± 16.2 (Tab. 3). A history of diabetes was reported by 17% of all men (women 10%). Almost 30% of the male and 24% of the female German population attending a GP were active smokers. The overall unemployed rate was 10.2%, the same as the official unemployment rate at October 2005 in Germany (10.4% Federal Statistic Office), indicating a good representativeness of the study population to the general German population with regard to social status.

**Table 3:** Patients characteristics stratified by sex in GEMCAS

	**Mean ± SD**	
	
	**Men** N = 13,942	**Women** N = 21,927
**Age (years)**	53.0 ± 15.8	50.9 ± 16.2
**BMI (kg/cm**^2^**)**	27.6 ± 4.4	26.6 ± 5.6
**Waist circumference (cm)**	98.8 ± 13.0	86.8 ± 14.3
**Blood pressure (mmHg)**		
Systolic	133.6 ± 18.2	128.5 ± 19.3
Diastolic	81.4 ± 10.4	79.2 ± 10.6
**Blood sample**		
Total cholesterol	203 ± 42.4	209 ± 40.8
HDL cholesterol	54.3 ± 14.2	67.3 ± 17.2
LDL cholesterol	128.1 ± 36.6	128.0 ± 36.6
Random blood glucose (mg/dL)	103 ± 36.5	94.7 ± 28.6
8 h fasting blood glucose (mg/dL)	99.7 ± 27.3	93.3 ± 23.6
Random triglycerides (mg/dL)	179.4 ± 164.6	135.2 ± 83.3
12 h fasting triglycerides (mg/dL)	166.3 ± 222.1	129.7 ± 74.3

	**N (%) **	
	
**School years**		
< 10 years	1205 (8.9)	3927 (18.4)
= 10 years	6914 (50.8)	11993 (56.2)
> 10 years	5481 (40.3)	5423 (25.4)
**Employment**		
Employed	7130 (52.2)	9726 (45.1)
Unemployed	977 (7.1)	933 (4.3)
Economically inactive^b^	5564 (40.7)	10901 (50.6)

**Diabetes mellitus**		
Type 1	125 (1.0)	99 (0.5)
Type 2	2.197 (16.6)	21.20 (10.1)
**History of Cardiovascular Disease**		
Myocardial infarction	1 504 (11.3)	736 (3.54)
Stroke	488 (3.7)	420 (2.0)
**Smoker**		
Yes	3 731 (27.5)	4 993 (23.5)
No, quit	5 395 (39.7)	4 739 (22.3)
No, never	4 445 (32.8)	11 501 (54.2)
**Sport activities**		
At least 2 h/week	3 528 (26.0)	4 984 (23.3)
Up to 2 h/week	4 886 (36.0)	8 730 (40.9)
No	5 178 (38.2)	7 640 35.8)

^a ^i.e. pupils, students, parents at leave, retirees

### Prevalence of the Metabolic Syndrome by NCEP ATP III 2001

**Figure 1:**
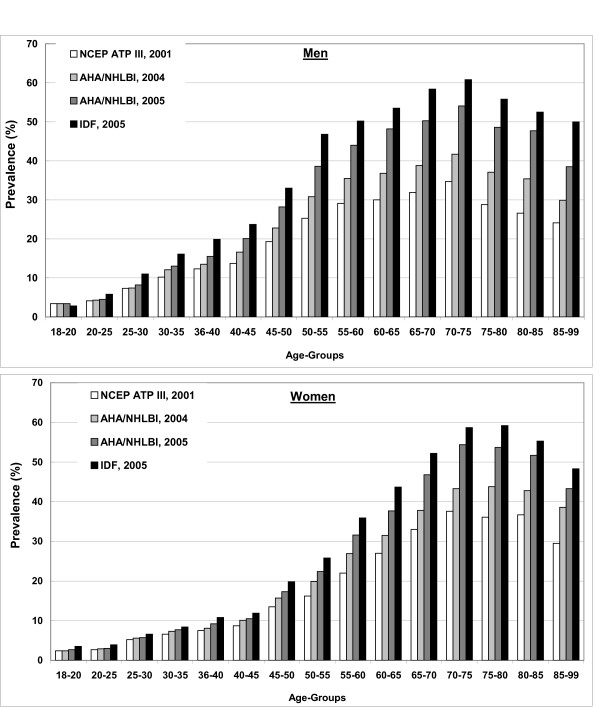
Overview of the distribution of prevalence estimates of the Metabolic Syndrome by age-group and sex according to different definitions.

The crude prevalence for the whole study sample was according to the most widely used definition NCEP ATP III (2001) 19.8% (men 22.7%, women 18.0%), age-standardized according to the German population 18.7%, (19.5%,18.05). The prevalence of the MetSyn increased with increasing age up to 70–75 years and was higher among men than among women up to the age of 65 years (figure 1). In patients >70 years, women were more often diagnosed than men.

MetSyn was diagnosed considerably more frequently among patients with <10 years of school education, with an higher difference found in women (Tab. 4). The age-specific prevalence in the lowest educational level was 1.7- to 3.0-fold higher in women (men: 1.5- to 2.0-fold) when compared to patients with >10 years of schooling.

**Table 4:** Prevalence of the Metabolic Syndrome in GEMCAS according to NCEP ATP III (2001) and IDF (2005) by education status (school years), age-group and sex

	**Age-Group**								
	
	**18–34**			**35–59**			**60–99**		
	
**School** **Years**	ATP III 2001	IDF 2005	IDF/NCEP	ATP III 2001	IDF 2005	IDF/NCEP	ATP III 2001	IDF 2005	IDF/NCEP
	**Men (n = 12,341)**								
<10	9.5%	10.8%	1.14	30.0%	46.7%	1.56	40.2%	61.3%	1.52
= 10	7.5%	11.1%	1.48	24.5%	40.8%	1.67	31.6%	58.8%	1.86
>10	5.5%	9.3%	1.69	15.4%	30.5%	1.98	26.5%	52.5%	1.98

<10/>10	1.7	1.2		2.0	1.5		1.5	1.2	

	**Women (n = 19,979)**								
<10	8.8%	10.1%	1.15	25.1%	35.0%	1.39	40.8%	61.1%	1.50
= 10	4.8%	6.4%	1.33	14.1%	21.7%	1.54	29.2%	48.9%	1.67
>10	3.5%	4.7%	1.34	8.3%	14.1%	1.70	24.0%	42.4%	1.77

<10/>10	2.5	2.1		3.0	2.5		1.7	1.4	

### Prevalence of the Metabolic Syndrome according to different definitions

The age-standardized prevalence of the whole study sample was lowest when assessed using the NCEP ATP III- (18.7%), highest using the IDF-definition (30.7%), (see Additional File 1). The pattern of distribution according to age and sex remains similar regardless of the definition used (Fig. 1).

**Figure 2:**
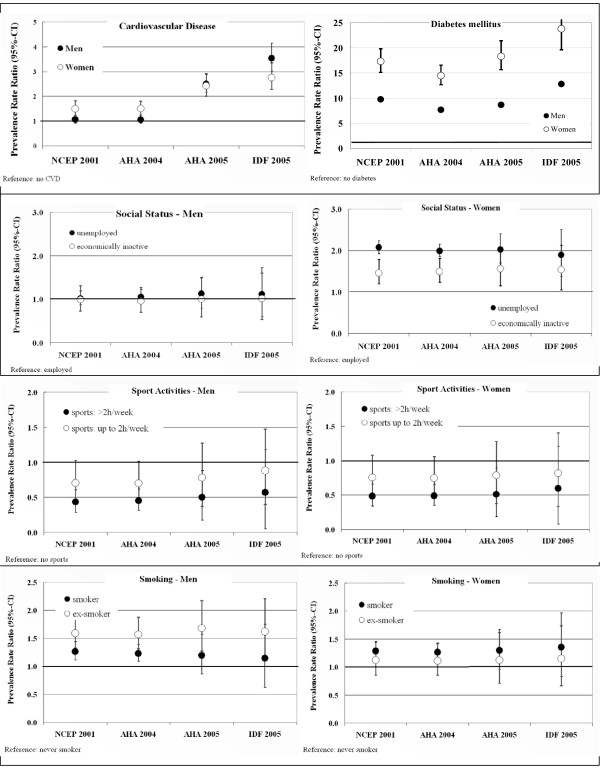
**Age-adjusted prevalence ratios for different definitions of the Metabolic Syndrome, stratified by sex**. For each definition the prevalence ratio was separately calculated, stratified by sex. Each model included the variables shown in this figure (CVD, diabetes, employment status, sport activities, smoking) as well as age, vine consumption, educational status. CVD and Diabetes mellitus were tested against not having CVD and Diabetes mellitus (reference), for social status employed subjects (reference) were compared with unemployed and economically inactive subjects.

Although in 2005 the AHA/NHLBI aligned their criteria with those of the IDF-definition for a better convergence, a difference in characterizing subjects as having the MetSyn is still apparent. We observed an absolute overall difference between these definitions of 4%, with a maximum of more than 7% in 50–59 year old men (see Additional File 1). However, the relative impact is highest for the youngest age-group, in both men and women. The level of agreement between the NCEP ATP-III-definition and the following definitions decreases with each newer definition proposed, with the lowest accordance to the IDF (see Additional File 1). The kappa between the newest AHA/NHLBI- and the IDF-definition (k 0.85, men 0.79, women 0.86) is still below the kappa between NCEP ATP III- and the AHA-definition released in 2004 (k 0.91). Fig. 2 shows that the tightened criteria of the IDF give rise to a higher chance (PRR) to be diagnosed with MetSyn in subjects who reported a history of myorcardial infarction, stroke or diabetes as compared to the other definitions. However, no differences seem to exist regarding risk factors like employment status, smoking or physically inactivity.

The absolute increase in prevalence was most extreme for men in the age-group 50–59, for women 60–69 (see Additional File 1). The impact of changed definitions is higher for men than women, widening the gender gap: 1.16-fold higher prevalence rates for men compared to women using the NCEP ATP III-definition, increasing to 1.20, 1.22 and 1.33 using the AHA/NHLBI-2004, 2005 and IDF-definition respective.

Regarding education, as proxy for socio-economic status (SES), use of the IDF-definition also resulted in a higher prevalence in all levels of education, with highest impact in those with the highest SES, slightly reducing the gap between levels of SES (Tab. 4). The overall PRR characterizing a person as having MetSyn according to years of schooling decreases in men with <10 years of schooling from 1.7 (95%-CI 1.5–1.9) according to NCEP-2001 to 1.3 (1.2–1.4), in women 2.2 (2.0–2.4) to 1.8 (1.6–1.9). A similar observation was made regarding the employment status (figure 2).

## Discussion

The results of this nationwide prevalence study show (1) a high prevalence of the metabolic syndrome in the German primary care population irrespective of the definition used, (2) large differences in prevalence rates when using different definitions, and (3) a varying impact of age, gender and SES on prevalence of metabolic syndrome between different definitions used.

The crude prevalence of the whole study population was 19.8% according to the most often used NCEP ATP III-, 23.5% according to AHA/NHLBI 2004-definition (age-standardized 18.7% and 22.0% respective). In line with previous published studies is the consistent observation of a high age dependence of the MetSyn, with a steady increase up to the age of 60 in some population in men [9] and 70 to 75 in both men and women in the US population [10], European populations [11,12] or in one Iran cohort [13].

Most countries reported slightly lower rates, like in an Irish primary care population [14], an Italian cohort [15] or a French [16] and Chinese working population [17], (see Additional File 2).

The prevalence in the US between 1988–1994 is slightly higher compared to GEMCAS (see Additional File 2). Ford and colleagues, using data from NHANES, estimated an age-adjusted prevalence of 23.7% based on a study sample of 8,814 participants [18]. With the same data set, but a reduced sample size (6,436), they reported two years later a prevalence of 24.1% [19]. Comparing data from NHANES 1988–1994 with newest NHANES data from 1999–2000, they reported a non-significant increase of the MetSyn among US adults up to 27.0% [19]. Unfortunately it remains recondite why one year later they estimated a prevalence of 34.5% with the same cohort [10]. Probably, different sample sizes ([19]: n = 1677, [10]: n = 3601) might account for these notable differences in the same cohort, although an indication for possible different sample selection is not provided. However, with these currently published prevalence data, a much higher proportion of the US adults seems to be classified as having the MetSyn compared to our German cohort.

In a sample of 3,589 British women free of CHD randomly selected from general practitioner lists [20], a prevalence with respect to the NCEP ATP III-definition of 29.8% was reported (see Additional File 2), which is much higher compared to 21.0% in GEMCAS women in the same age-range and free of CHD. However, Lawlor and colleagues modified the NCEP ATP III-definition by using the HDL-C threshold for men (<1 mmol/L instead of <1.3 mmol/L for women). Applying this threshold to GEMCAS, only 18.4% of the women would have been classified as having MetSyn.

Our data show the dependence of the estimated occurrence of the Metabolic syndrome on their definition used. However, not only different definitions result in different prevalence rates, but also crude applications and diverse – often not indicated – modifications of the original definitions exacerbate this situation (see Additional File 2). Conspicuous are on one hand minor changes to the definitions which refer to the use of "≥ " instead of just '>', i.e. [21]. Sometimes cut-offs even changed by differently rounding off in SI units (HDL: 39–40 mg/dL equal to 0.9–1.04 mmol/L or glucose 100–101 mg/dL equal to 5.6 mmol/L, triglyceride ≥ 1.69–1.71 mmol/L equal to 150 mg/dL) i.e. [11,22,23], which in our study changed the prevalence by 2% to almost 4% respectively. Furthermore, a common difference is found to be length of fasting period, reported as "overnight fast", a 6, 8, 12 or 14 hours to a >12 hours fasting period. Taking only these different time periods into account, we receive estimates in our GEMCAS sample ranging between 16–21%.

On the other hand, major changes are the inclusion of pharmacotherapy as criteria for hypertension or diabetes, i.e. [10,20,24-28]. Pharmacotherapy is not mentioned in the original NCEP ATP III- and AHA/NHLBI-definitions and explicitly not included until the 2005 AHA/NHLBI-update [4]. Besides, it remains unclear, why most of these studies included hypertensive medications, but not lipid lowering drugs. However, subjects who were treated for hypertension, hypertriglyceridemia, or diabetic in this study were not defined as hypertensive or diabetes when using the NCEP ATP-III definition from 2001 and 2004. It remains an open point of discussion, if this would create a selection bias leading to an under- or overestimation of the true prevalence of MetSyn, since the impact of the MetSyn on developing diabetes mellitus and CVD is still unclear. Furthermore, the question of drug therapy inclusion certainly depends on the application of the MetSyn, i.e. if primary prevention issues are of particular interest, than including treated subject does not make sense.

Further differences like study designs, selection of study populations, age ranges, measurement methods, and at latest missing age-standardized prevalence data, make comparisons of published prevalence rates rather difficult (see Additional File 2). Subsequently, the majority of studies reported a wide range of prevalence rates in the general population and it remains unresolved, if the differences between populations are real difference due to possibly specific genetic constituents, likes and dislikes in life styles or due to above mentioned methodological inconsistencies – or all together.

Regarding the difference in prevalence of the MetSyn between sexes, the picture is contrary to age blurred. In GEMCAS, prevalence is consistently higher in men in the age-range 20 to 70. Similar gender differences are found in Finland [29], Sweden [30], France [12], Italy [15] and in a pooled European population [31,32]. In contrast, higher rates in women were reported from Spain [25], Netherlands [33], Greenland (Inuit) [21], India [34] and West Bank [35], whereas almost equal proportions were reported from the US population [10,25].

Without scrutinizing the whole concept of the MetSyn – this has been done extensively, [36-38] to name only some – opinions about the adequate definition to assess the MetSyn are still controversial. Although in 2005 the IDF released a so called worldwide new definition, the AHA/NHLBI ensued only some month later with an own, modified one. In fact, the sets of clinical criteria of both definitions have converged. In their statement of the AHA and NHLBI they almost appeal, that despite "minor differences" both definitions now identify essentially the same individuals as having the MetSyn, at least in the US population [4]. This of course is not false, since almost all individuals identified as having the MetSyn with the modified AHA 2005-definition were also identified by the IDF-definition. In GEMCAS only 28 patients (0.1%) were diagnosed as having MetSyn with the AHA 2005-definition but not with the IDF-definition. But this is only the one aspect. The other aspect, using the IDF-definition, reveals about 4% more subjects classified as having the syndrome, even 7% in men aged 50–80 years, which in GEMCAS on its own accounts for an overall additional 1,600 subjects. The agreement (kappa) between the definitions is still less than 0.9.

The strengths of the study are the sample size, the nationwide approach and the assessment of the MetSyn as primary study target, so comprising all required variables as original measures. However, the participants are strictly speaking not a real population-based sample, but close to being so. Some patients meeting MetSyn criteria might avoid attending their physician because they are in denial, in this case an underestimation of the prevalence would have been occurred. On the other hand, the estimated prevalence might be too high because the healthy population does not routinely visit their physician. However, 91.8% of the adult persons in Germany consult a general practitioner during one year [39]. Furthermore, the sample is comparable to other German population-based samples i.e. with regard to anthropometric measures, smoking status, marital status and schooling. However, the proportion of participants with chronic diseases (diabetes mellitus, CVD) is higher than compared to population-based sample, but still lower than real patient-based samples [40]. Lastly, this study was conducted and analysed in line with the way procedures would have been performed if they had been taken place under real circumstances in clinical practices. This report is thus meaningful because it is the first to provide prevalence rates for the MetSyn as defined to different definition and to different subgroups of the population.

## Conclusion

These epidemiological data indicate a high prevalence of the metabolic syndrome in the German population attending a general practitioner. Especially high rates were found in older subjects and subjects less well educated. Concerning prevention issues, the primary scope of application of the MetSyn is claimed to be prevention of CVD and diabetes mellitus. Thus it is questionable to what extent the most recent definitions are helpful to identify persons at risk without falsely labelling to much people to be at risk [41-43], with the postulated consequence of a rapid and aggressive treatment [5]. Our data highlight the need for a better evidence regarding thresholds of the components of the MetSyn, especially with regard to the IDF-definition – according to which in some subpopulations a majority of subjects are diagnosed with the metabolic syndrome. This could be accomplished by the use of epidemiological, longitudinal data.

## Competing interests

All listed authors declare that there were no competing interests involved with this research.

## Authors' contributions

SM planned and performed the study and wrote the manuscript. JH participated in the design of survey instruments and performed the statistical analysis. PB has been revising the manuscript critically for important intellectual content, JW participated in the study design, PA coordinated administrative issues of the project. KHJ supervised scientific, ethical and data privacy issues of the study. All authors read and approved the final manuscript.

## Supplementary Material

Additional file 1 Prevalence of the Metabolic Syndrome according to different definitions by age and sex. The data provided give a detailed overview of the metabolic syndrome prevalence according to age and gender. Click here for file

Additional file 2 Prevalence estimates of the Metabolic Syndrome of selected studies compared to the GEMCAS population. The data provided give an overview about selected studies worldwide and their comparison to the GEMCAS population. Click here for file
