# Supplementary material for: Impact of 4 different definitions used for the assessment of the prevalence of the Metabolic Syndrome in primary healthcare:The German Metabolic and Cardiovascular Risk Project (GEMCAS)
Source: Cardiovasc Diabetol. 2007 Sep 6;6:22. doi: 10.1186/1475-2840-6-22 (PMC2031874; doi:10.1186/1475-2840-6-22)
Supplement: Additional file 1 — Prevalence of the Metabolic Syndrome according to different definitions by age and sex. The data provided give a detailed overview of the metabolic syndrome prevalence according to age and gender. [file 1475-2840-6-22-S1.pdf]

Table 5 - Prevalence of the Metabolic Syndrome in GEMCAS according to different definitions by age and sex

				**Changes compared to NCEP/ATP-III:** hypertensive treatment included		glucose cut off reduced		drug treatment introduced		Increased waist required and waist cut off reduced			
**Age-Group**	**Total N**	**NCEP/ATP-III 2001** %	**SD**	**NCEP/ATP-III mod.**[1] %	**SD**	**AHA/NHLBI 2004**[*] %	**SD**	**AHA/NHLBI 2005**[*] %	SD	**IDF 2005** %	**SD**	**IDF/AHA 2005**	**Δ**[†] %
**All**													
18-29	3541	4.3	0.20	4.4	0.21	4.5	0.21	4.8	0.21	5.9	0.24	1.23	1.11
30-39	4472	8.6	0.28	9.0	0.29	9.5	0.29	10.6	0.31	12.7	0.33	1.20	2.15
40-49	7173	13.1	0.34	14.0	0.35	15.3	0.36	17.5	0.38	20.2	0.40	1.16	2.73
50-59	7404	22.4	0.42	24.7	0.43	27.4	0.45	32.8	0.47	38.0	0.49	1.16	5.18
60-69	5586	30.5	0.46	33.1	0.47	36.2	0.48	45.6	0.50	51.8	0.50	1.14	6.26
70-79	4017	34.9	0.48	38.6	0.49	41.9	0.49	53.1	0.50	58.9	0.49	1.11	5.73
80-99	1116	31.9	0.47	36.3	0.48	39.2	0.49	48.4	0.50	53.0	0.50	1.10	4.67
**total**	**33502**	**19.8**	**0.40**	**21.6**	**0.41**	**23.5**	**0.42**	**28.6**	**0.45**	**32.7**	**0.47**		
**total** [‡]		**18.7**	**0.54**	**20.3**	**0.55**	**22.0**	**0.57**	**26.9**	**0.61**	**30.7**	**0.63**	**1.14**	**4.07**
$\kappa^{\S}$				0.96		0.91		0.80		0.68			
$\kappa^{\|}$										0.85			
**Men**													
18-29	1197	5.4	0.23	5.4	0.23	5.5	0.23	6.0	0.24	7.6	0.27	1.28	1.64
30-39	1510	11.5	0.32	12.0	0.33	13.0	0.34	14.5	0.35	18.3	0.39	1.27	3.87
40-49	2471	16.8	0.37	17.8	0.38	19.9	0.40	24.5	0.43	28.7	0.45	1.17	4.23
50-59	2974	27.3	0.45	29.9	0.46	33.4	0.47	41.5	0.49	48.7	0.50	1.17	7.14
60-69	2463	31.0	0.46	34.0	0.47	37.9	0.49	49.4	0.50	56.3	0.50	1.14	6.95
70-79	1771	32.2	0.47	36.4	0.48	39.8	0.49	51.9	0.50	58.8	0.49	1.13	6.88
80-99	383	26.1	0.44	31.4	0.46	34.2	0.48	45.8	0.50	52.0	0.50	1.14	6.19
**total**	**12838**	**22.7**	**0.42**	**24.9**	**0.43**	**27.5**	**0.45**	**34.8**	**0.48**	**40.3**	**0.49**		
**total**[‡]		**19.5**	**0.61**	**21.2**	**0.63**	**23.3**	**0.65**	**29.2**	**0.70**	**34.1**	**0.73**	**1.16**	**5.57**
$\kappa^{\S}$				0.96									
$\kappa^{\|}$										0.79			

				**Changes compared to NCEP/ATP-III:** hypertensive treatment included		glucose cut off reduced		drug treatment introduced		Increased waist required and waist cut off reduced			
**Age-**		**NCEP/ATP-III 2001**		**NCEP/ATP-III mod.**[1]		**AHA/NHLBI 2004***		**AHA/NHLBI 2005***		**IDF 2005**		**IDF/ AHA 2005**	**Δ†**
**Group**	**Total N**	**%**	**SD**	**%**	**SD**	**%**	**SD**	**%**	SD	**%**	**SD**		**%**
**Women**													
18-29	2344	3.8	0.19	3.9	0.19	4.0	0.20	4.2	0.20	5.0	0.22	1.20	0.84
30-39	2962	7.2	0.26	7.5	0.26	7.8	0.27	8.6	0.28	9.8	0.30	1.15	1.25
40-49	4702	11.1	0.31	12.1	0.33	12.9	0.33	13.8	0.35	15.8	0.36	1.14	1.97
50-59	4430	19.1	0.39	21.2	0.41	23.4	0.42	27.0	0.44	30.8	0.46	1.14	3.87
60-69	3123	30.2	0.46	32.4	0.47	34.9	0.48	42.6	0.49	48.3	0.50	1.13	5.7
70-79	2246	36.9	0.48	40.4	0.49	43.5	0.50	54.1	0.50	58.9	0.49	1.09	4.84
80-99	733	35.0	0.48	38.9	0.49	41.8	0.49	49.7	0.50	53.6	0.50	1.08	3.89
**total**	**20664**	**18.0**	**0.38**	**19.6**	**0.40**	**21.0**	**0.41**	**24.8**	**0.43**	**28.0**	**0.45**		
**total‡**		**18.0**	**0.49**	**19.5**	**0.50**	**20.9**	**0.51**	**24.8**	**0.55**	**27.7**	**0.57**	**1.13**	**3.12**
$\kappa^{\S}$				0.96		0.92		0.83		0.74			
$\kappa^{\|}$										0.89			

[1] Modification of the original NCEP/ATP-III-definition, considering subjects using hypertensive medication as having MetSyn

* often cited as NCEP ATP III-definition

† AHA 2005 - IDF 2005

‡ age standardized according to German population (destatis 2005)

§ kappa of accordance between prevalence of NCEP/ATP-III and following definitions

|| kappa of accordance between prevalence of AHA/NHLBI (2005) and IDF-definition
