# Supplementary material for: Impact of 4 different definitions used for the assessment of the prevalence of the Metabolic Syndrome in primary healthcare:The German Metabolic and Cardiovascular Risk Project (GEMCAS)
Source: Cardiovasc Diabetol. 2007 Sep 6;6:22. doi: 10.1186/1475-2840-6-22 (PMC2031874; doi:10.1186/1475-2840-6-22)
Supplement: Additional file 2 — Prevalence estimates of the Metabolic Syndrome of selected studies compared to the GEMCAS population. The data provided give an overview about selected studies worldwide and their comparison to the GEMCAS population. [file 1475-2840-6-22-S2.pdf]

Table 6 - Prevalence estimates of the metabolic syndrome of some published studies compared to the GEMCAS population

Country	Study name	Study cohort	Sample size	Age range	Definition	Prevalence %	GEMCAS, % (n) *	Reference
Korea	-	medical check-up based, Seoul + Kyung-Gi province	40,698	20-82	NCEP 2001 + medication	6.8 (10.9) †	20.2	[26]
China	InterASIA	nationwide	15,540	35-74	NCEP 2001 + medication	13.7 (15.8) †	23.3 (24,885)	[28]
Ireland	-	primary care, metropolitan (Cork and Kerry)	1,018	50-69	NCEP 2001	20.7	25.9 (12,432)	[14]
Italy	PAMELA	metropolitan (Monza)	2,013	25-74	(?)	16.2	19.7/21.5‡ (28,610)	[15]
France	-	working cohort		30-65	NCEP 2001	9.8	13.4 (13,277)	[16]
China, Hong Kong	-	metropolitan, working	1,513	18-66	NCEP 2001 + medication	9.6	12.0 (15,963)	[17]
Iran	Tehran Lipid and Glucose	metropolitan (Tehran)	10,368	>20	NCEP 2001	30.1	19.0	[13]
Great Britain	British Women's Heart and Health	general practices, metropolitan (23 British towns)	4,286	60-79	NCEP 2001 + medication	29.8	21.0 (18,4) §	[20]
Oman	Nizwa Healthy Life Style Project Survey	metropolitan (Nizwa)	1,419	>20	NCEP 2001 + medication	21.0	19.7	[9]
Greece	The MetS-Greece Multicentre	nationwide	4,153	>18	NCEP 2001 + medication	23.6	21.5	[11]
US	NHANES III (1988-1994)	nationwide	8,814	>20	NCEP 2001 + medication	23.7 (24.1) ‖	20.3	[18]
	NHANES (1999-2000)	nationwide	1,677	>20	NCEP 2001 + medication	27.0 (34.5) #	20.3	[19]

* prevalence of the GEMCAS cohort estimated according to age range, sex employment status of the specific study population

† when an ethnicity adapted measure for obesity was used

‡ depending on definition used (NCEP 2000/AHA 2004)

§ these differences are even higher, since Lawlor et al. [20] modified the original NCEP-definition by using the HDL-C threshold for men -(<1mmol/L instead of <1.3mmol/L for women). Applying this threshold to GEMCAS, only 18.4% of the women would have been classified as having MetSyn

‖ data in ( ) from same data set, but different publication [10]

\# published in [10], probably different prevalences because of different sample sizes (n=3,601)
